# Effects of combined cognitive and physical intervention on enhancing cognition in older adults with and without mild cognitive impairment: A systematic review and meta-analysis

**DOI:** 10.3389/fnagi.2022.878025

**Published:** 2022-07-19

**Authors:** Kaiyue Han, Zhiqing Tang, Zirong Bai, Wenlong Su, Hao Zhang

**Affiliations:** ^1^School of Rehabilitation, Capital Medical University, Beijing, China; ^2^China Rehabilitation Research Center, Beijing Bo'ai Hospital, Beijing, China; ^3^Faculty of Medicine, Dentistry and Health Sciences, University of Melbourne, Parkville, VIC, Australia; ^4^University of Health and Rehabilitation Sciences, Qingdao, China; ^5^Cheeloo College of Medicine, Shandong University, Jinan, China

**Keywords:** combined cognitive and physical intervention, cognition, older adults, mild cognitive impairment, systematic review, meta-analysis

## Abstract

**Background:**

Combined cognitive and physical intervention is commonly used as a non-pharmacological therapy to improve cognitive function in older adults, but it is uncertain whether combined intervention can produce stronger cognitive gains than either single cognitive or sham intervention. To address this uncertainty, we performed a systematic review and meta-analysis to evaluate the effects of combined intervention on cognition in older adults with and without mild cognitive impairment (MCI).

**Methods:**

We systematically searched eight databases for relevant articles published from inception to November 1, 2021. Randomized controlled trials (RCTs) and non-randomized controlled trials (NRCTs) were used to compare the effects of the combined intervention with a single cognitive or sham intervention on cognition in older adults with and without MCI aged ≥ 50 years. We also searched Google Scholar, references of the included articles, and relevant reviews. Two independent reviewers performed the article screening, data extraction, and bias assessment. GRADEpro was used to rate the strength of evidence, and RevMan software was used to perform the meta-analysis.

**Results:**

Seventeen studies were included in the analysis, comprising eight studies of cognitively healthy older adults and nine studies of older adults with MCI. The meta-analysis showed that the combined intervention significantly improved most cognitive functions and depression (SMD = 0.99, 95% CI 0.54–1.43, *p* < 0.0001) in older adults compared to the control groups, but the intervention effects varied by cognition domains. However, there was no statistically significant difference in the maintenance between the combined and sham interventions (SMD = 1.34, 95% CI −0.58–3.27, *p* = 0.17). The subgroup analysis also showed that there was no statistical difference in the combined intervention to improve global cognition, memory, attention, and executive function between cognitive healthy older adults and older adults with MCI.

**Conclusions:**

Combined intervention improves cognitive functions in older adults with and without MCI, especially in global cognition, memory, and executive function. However, there was no statistical difference in the efficacy of the combined intervention to improve cognition between cognitive healthy older adults and older adults with MCI. Moreover, the maintenance of the combined intervention remains unclear due to the limited follow-up data and high heterogeneity. In the future, more stringent study designs with more follow-ups are needed further to explore the effects of combined intervention in older adults.

**Systematic review registration:**

https://www.crd.york.ac.uk/PROSPERO/#recordDetails, identifier: CRD42021292490.

## Introduction

As the global population ages, cognitive decline has become an increasingly critical factor affecting the health and quality of life of older adults, ranging from normal cognitive function to mild cognitive impairment (MCI) even dementia (Anderson, 2020). In recent years, the prevalence of MCI has increased in older adults, exacerbating the potential impact on global physical and mental health (Vos et al., 2015; Overton et al., 2019). A study has shown that the proportion of participants with depression among older adults with MCI ranged from 20.1 to 44.3% (Panza et al., 2010), and improvement in this state of MCI plus depression (MCI/D) is an essential factor in improving quality of life. MCI is an early stage of memory loss or other cognitive ability loss in individuals who maintain the ability to independently perform most activities of daily living (ADL) (Jack et al., 2018). Moreover, MCI has a high risk of progressing into Alzheimer's disease (AD) and other dementias, with reported conversion rates of 50% in 2-3 years (Marioni et al., 2015) and even as high as 60–100% in 5–10 years (Albert et al., 2011).

MCI refers to a cognitive and functional decline syndrome with no currently available cure. At present, pharmacological treatments for patients with MCI have not been proven to be completely effective, and adverse effects have been observed (Briggs et al., 2016). Cognitive interventions using non-invasive and non-pharmacological treatments based on the theories of neuroplasticity (Greenwood and Parasuraman, 2010; Rajji, 2019) and rich environments have attracted more attention (Marlats et al., 2020; Liu et al., 2021). A previous study reported that older adults with and without MCI showed signs of cognitive decline to varying degrees, and combined cognitive and physical intervention effectively improves cognition (Wu et al., 2019), which also becomes a research hotspot in recent years. Shatil (2013) conducted a 16-week randomized controlled trial (RCT) of combined cognitive and physical intervention, single cognitive intervention, and sham intervention in 29, 33, and 29 cognitively healthy older adult subjects, respectively, and found that combined intervention was significantly better than single cognitive intervention in improving memory and naming, while sham intervention showed no improvement in cognition. Additionally, Park et al. (2019) conducted a 24-week RCT in 49 older adult subjects with amnesic MCI (aMCI), in which 25 subjects performed aerobic exercise while doing number crunching and found that combined intervention improved working memory and executive function, but the sham intervention did not improve cognition in the other 24 subjects.

Although many meta-analyses have reported the cognitive benefits of the combined intervention for older adults with and without MCI (Stanmore et al., 2017; Gheysen et al., 2018; Gavelin et al., 2021), they were mixed across age groups and included articles that varied considerably in terms of study designs, comparisons, and study qualities. Therefore, the efficacy of the combined intervention to improve cognition is yet to be determined, especially when compared to single cognitive intervention (Law et al., 2014; Wollesen et al., 2015; Zhu et al., 2016). To address the above limitations, this meta-analysis developed a more detailed inclusion criteria and separately reported the effects of the combined intervention compared with a single cognitive or sham intervention on cognition in older adults with and without MCI.

The objectives of this systematic review and meta-analysis are as follows: (1) to compare the effects of combined intervention with a single cognitive or sham intervention on cognition in older adults; (2) to explore the differences in cognitive efficacy of the combined intervention for cognitively healthy older adults and those with MCI; and (3) to summarize and compare the maintenance and safety of combined intervention in order to provide practical strategies and methods for improving cognition in older adults.

## Methods

We report the systematic review and meta-analysis following the Preferred Reporting Items for Systematic Reviews and Meta-analysis guidelines (Moher et al., 2009) and register the review in the International Prospective Register of Systematic Reviews (CRD42021292490).

### Search strategy

We implemented the search strategy by using a combination of MESH terms, free-text words, and truncation retrieval, and we searched for articles on combined cognitive and physical intervention to enhance cognition in older adults with and without MCI published in PubMed, Embase, Web of Science, Cochrane Library, PsycINFO, Scopus, EBSCO and Ovid from inception to November 1, 2021. Furthermore, we screened all reference lists of the selected articles and related review articles, and we used the same search terms in Google Scholar to perform additional searches. The search was limited to publications in English. The complete search strategy (Supplementary Table S1) is provided in the Supplementary Material.

### Selection criteria

The inclusion criteria of this meta-analysis is detailed below.

#### Participants

Studies were included if the participants: were cognitively healthy older adults or those diagnosed with MCI; had an age of 50 years or older.

#### Interventions

Combined cognitive and physical training as an intervention that is either a simultaneous or a sequential dual or multi-tasking (Gallou-Guyot et al., 2020), refers to performing two or even more cognitive and physical tasks separately or simultaneously (Tait et al., 2017; MacPherson, 2018). We did not limit the cognitive or physical training type in the combined intervention.

#### Comparisons

The intervention in the control group included either single cognitive or sham intervention (e.g., placebo control, blank control, and passive control) for older adults with or without MCI.

If the study had two or more control groups (e.g., single physical intervention, single cognitive intervention, or sham intervention), only data from the control group with single cognitive or sham intervention were included.

#### Outcomes

The primary outcome was cognitive function, including global cognitive function, memory, attention, and executive function; the secondary outcome was depression.

##### Cognition evaluation

Mini-Mental State Examination (MMSE) and the Montreal Cognitive Assessment (MoCA) to evaluate the global cognition; Logical Memory (LM), Digit Span Test (DST), Trail Making Test Parts A (TMT-A), Rey Auditory Verbal Learning Test (RAVLT), and Complex Figure Test (CFT) to evaluate memory function; Symbol Digit Substitution Test (SDST), Brief Test of Attention (BTA), Test of Everyday Attention (TEA), and attentional Matrices (AM) to evaluate attention; Trail Making Test Parts B (TMT-B) and Executive Function Cognitive Assessment Scale (FUCAS) to assess executive function; Stroop color-word test (SCWT) to evaluate inhibition and executive control function.

##### Depression evaluation

The included studies used the Geriatric Depression Scale (GDS) or the Cornell Scale for Depression in Dementia (CSDD) to assess depression.

#### Design

Studies that were randomized controlled trials (RCTs) or non-randomized controlled trials (NRCTs) were included in this review.

### Study selection and data extraction

Two reviewers (HKY, TZQ) worked independently to screen the articles, extract information, and cross-check. In case of a disagreement, the articles were reviewed by a third reviewer (SWL). The authors of the original study were contacted *via* email to clarify or add any missing information. The articles were initially screened by reading the title and abstract before reading of the full text for re-screening. For each eligible study, we used a self-designed standardized form (Supplementary Table S2) to extract the first author's name, year of publication, country, clinical diagnosis of disease, number of participants, male ratio, age, education level, intervention methods, intervention characteristics, outcome measures, and drop-out.

### Risk of bias and study quality assessment

Two reviewers (HKY, TZQ) independently assessed the studies according to the Cochrane Handbook for Systematic Reviews of Interventions (Higgins et al., 2011), and disagreements on assessments were resolved by discussion with the third reviewer (SWL). The assessment scale included the following seven items: random sequence generation and allocation concealment (selection bias), blinding of participants and personnel (performance bias), blinding of outcome assessment (detection bias), incomplete outcome data (attrition bias), selective reporting (reporting bias), and other bias. Three degrees of assessment were used to grade each item: “low,” “unclear,” and “high.”

The PEDro scale, comprising 11 items, was used to assess the quality of the included studies, and studies with a score of seven or higher were considered to be of medium and high quality (Maher et al., 2003). Based on the risk of bias, inconsistency, indirectness, imprecision, and publication bias, the online GRADEpro method was used to evaluate the quality of evidence for pooled results in the meta-analysis (Cui et al., 2019).

### Data analysis and statistical methods

We used RevMan software 5.4 to perform the meta-analysis. Since all data were continuous information and were pooled by the same outcome using inconsistent scales, we selected the Standardized Mean Difference (SMD) as an effective indicator and provided the 95% confidence interval (CI). We used the Cochrane *Q* statistic to qualitatively determine whether heterogeneity existed among the included studies (test level α = 0.05), while the *I*^2^ statistic was used to quantitatively determine the magnitude of heterogeneity. If the *P-*value was ≥ 0.1 and *I*^2^ ≤ 50%, the heterogeneity was considered to be insignificant and we selected the fixed-effects (FE) model. Conversely, if the heterogeneity was considered to be significant, we selected the random-effects (RE) model and performed a subgroup analysis and sensitivity analysis to identify the factors that contributed to the heterogeneity. Descriptive analysis was performed if the source of heterogeneity could not ultimately be determined.

## Results

### Study selection

**Figure 1:**
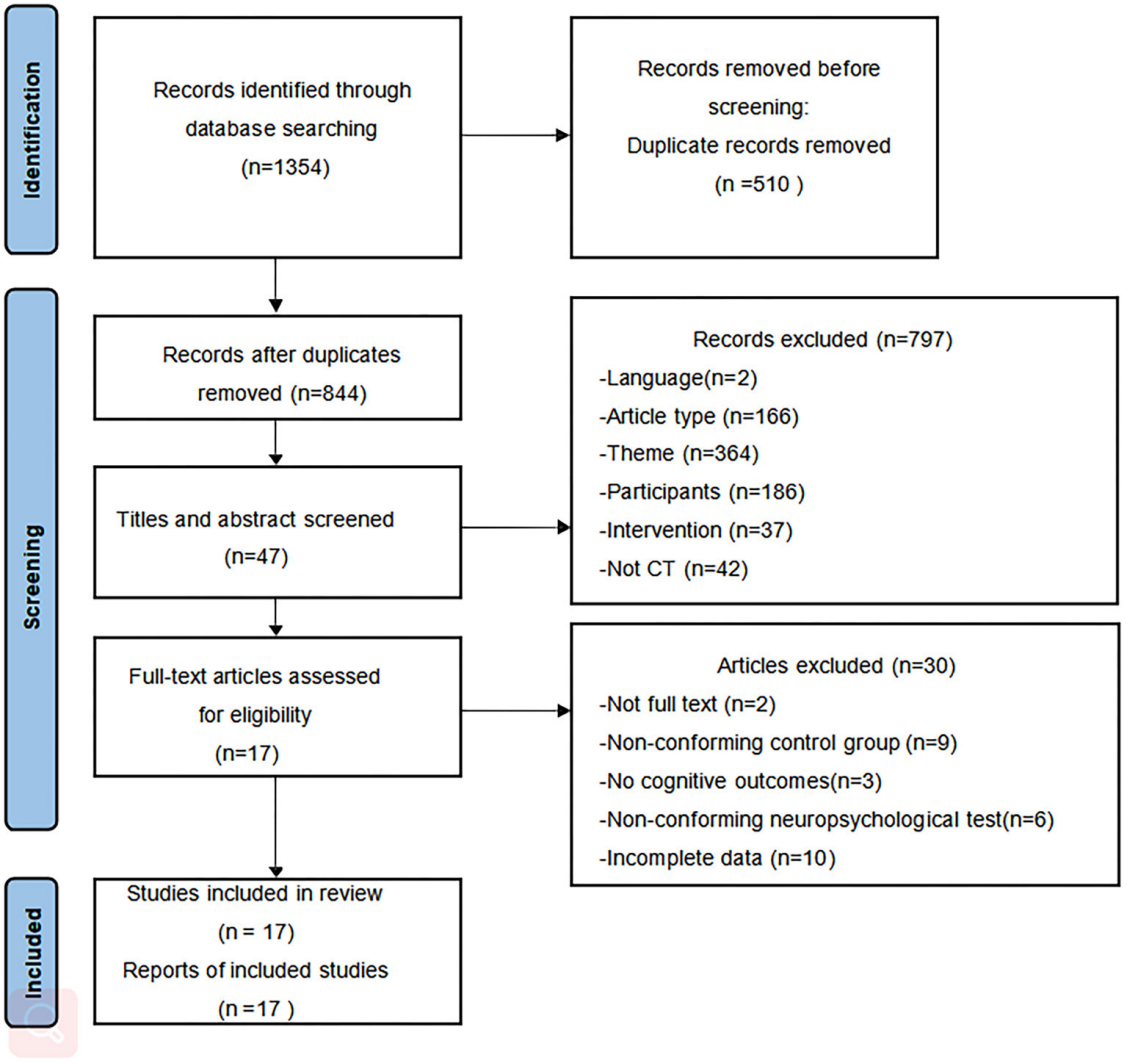
PRISMA flowchart of study selection.

The flowchart of study selection is shown in Figure 1. We initially retrieved 1,353 articles from the nine databases and identified one article through other sources. Eight hundred and forty-four articles remained after removing duplicates using a reference management software. After reading the titles and abstracts for screening, 797 articles were excluded. Subsequently, after screening the full text of the remaining 47 articles, 10 articles were not full data available, nine articles had a non-conforming control group, three articles had no cognitive assessment results, six articles had no conforming neuropsychological tests, and the full text of two articles were not available. Finally, 17 articles were included in this review.

### Characteristics of the included studies

As shown in Supplementary Table S2, eight studies of cognitively healthy older adults were eligible (Fabre et al., 2002; Marmeleira et al., 2009; Shatil, 2013; Hars et al., 2014; Nishiguchi et al., 2015; Rahe et al., 2015a,b; Morita et al., 2018), with 181 participants in the combined intervention group, 68 in the single cognitive intervention group, and 142 in the sham intervention group. Regarding the study design, six studies were RCTs (Fabre et al., 2002; Marmeleira et al., 2009; Shatil, 2013; Hars et al., 2014; Nishiguchi et al., 2015; Rahe et al., 2015a) and two studies were NRCTs (Rahe et al., 2015b; Morita et al., 2018). Regarding the modes of combined intervention, four studies performed simultaneous combined cognitive and physical training (Marmeleira et al., 2009; Shatil, 2013; Hars et al., 2014; Nishiguchi et al., 2015; Morita et al., 2018) and four studies performed sequential combined intervention (Fabre et al., 2002; Shatil, 2013; Rahe et al., 2015a,b), all of which reported greater cognitive gains in the combined intervention. Regarding the comparison condition, three studies used single cognitive intervention (Shatil, 2013; Rahe et al., 2015a,b), one study used reading as a placebo control (Shatil, 2013), four studies used a blank control (Fabre et al., 2002; Marmeleira et al., 2009; Hars et al., 2014; Nishiguchi et al., 2015), and one study used non-exercise as a passive control (Morita et al., 2018). Additionally, five studies implemented interventions longer than 12 weeks (Marmeleira et al., 2009; Shatil, 2013; Hars et al., 2014; Nishiguchi et al., 2015; Morita et al., 2018). Only one study had a follow-up - up to 1 year -and reported that combined intervention can produce more significant long-term effects than single cognitive intervention, especially in attention (Rahe et al., 2015b).

Nine studies of older adults with MCI were eligible (Kounti et al., 2011; Lam et al., 2015; Delbroek et al., 2017; Park, 2017; Donnezan et al., 2018; Mrakic-Sposta et al., 2018; Park et al., 2019, 2020; Rojasavastera et al., 2020), with 217 participants in the combined intervention group, 41 in the single cognitive intervention group, and 176 in the sham intervention group. Regarding the study design, eight studies were RCTs (Lam et al., 2015; Delbroek et al., 2017; Park, 2017; Donnezan et al., 2018; Mrakic-Sposta et al., 2018; Park et al., 2019, 2020; Rojasavastera et al., 2020) and one study was NRCT (Kounti et al., 2011). Regarding the modes of combined intervention, seven studies included simultaneous combined cognitive and physical training (Kounti et al., 2011; Delbroek et al., 2017; Park, 2017; Donnezan et al., 2018; Mrakic-Sposta et al., 2018; Park et al., 2019, 2020) and two studies performed sequential combined intervention (Lam et al., 2015; Rojasavastera et al., 2020), all of which reported greater cognitive improvements in the combined intervention. Regarding the comparison condition, three studies used single cognitive intervention (Park, 2017; Donnezan et al., 2018; Park et al., 2020), one study used social activities as a placebo control (Lam et al., 2015), and five studies used a blank control (Kounti et al., 2011; Delbroek et al., 2017; Mrakic-Sposta et al., 2018; Park et al., 2019; Rojasavastera et al., 2020). Additionally, four studies implemented interventions longer than 12 weeks (Kounti et al., 2011; Lam et al., 2015; Donnezan et al., 2018; Park et al., 2019). Only three studies had follow-up—up to 1, 3, and 6 months, respectively—and they also reported greater long-term cognitive improvements in combined intervention group (Donnezan et al., 2018; Park et al., 2019; Rojasavastera et al., 2020).

### Risk of bias and quality assessment

**Figure 2:**
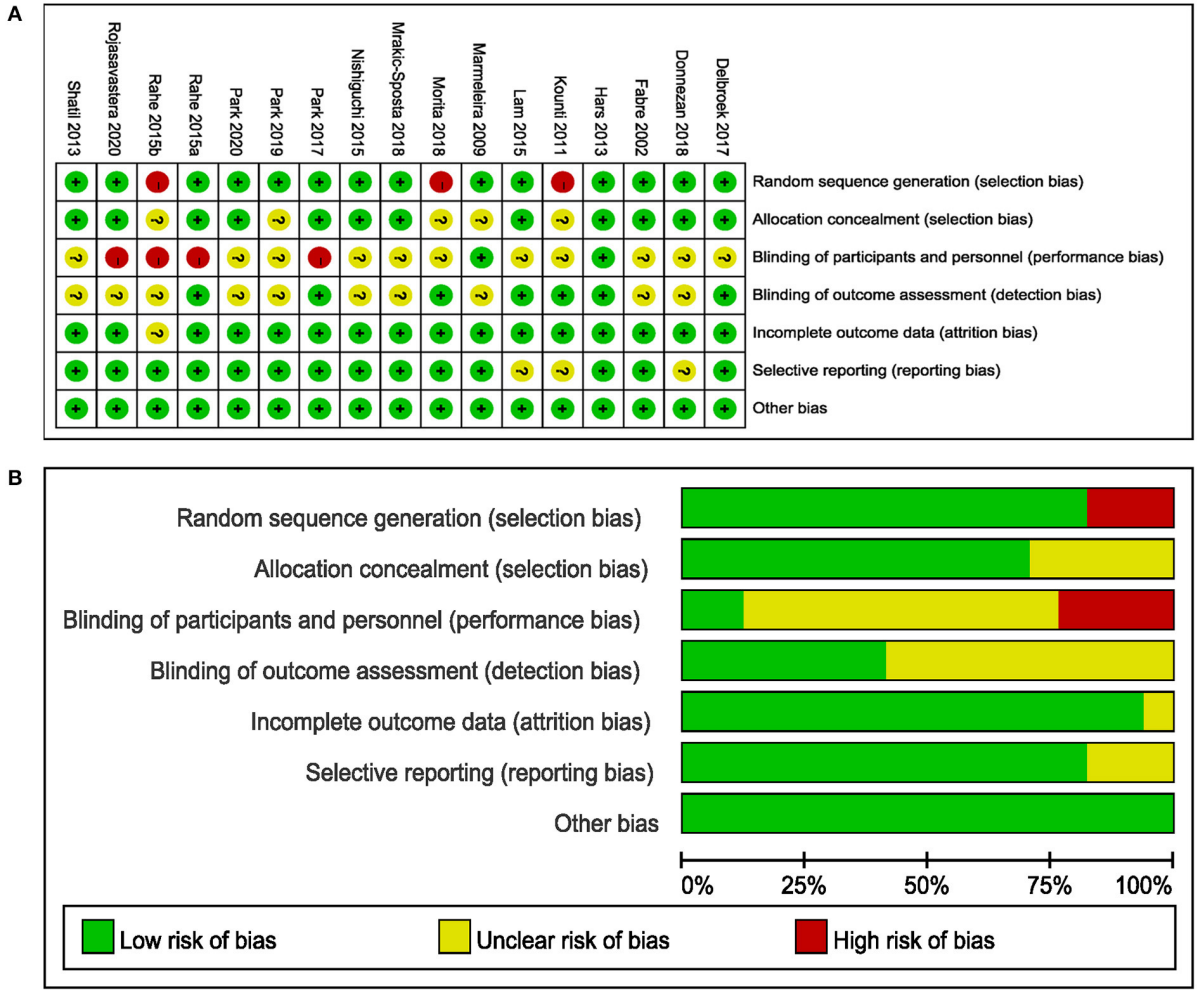
Results from the Cochrane risk of bias (ROB) tool. **(A)** ROB graph and **(B)** ROB summary.

The PEDro scale showed that all studies were non-low quality (Supplementary Table S2). The risk of bias of the included studies is shown in Figure 2. Of the 17 studies included, three studies did not use randomization methods (Kounti et al., 2011; Rahe et al., 2015b; Morita et al., 2018) and four did not report allocation concealment (Marmeleira et al., 2009; Kounti et al., 2011; Morita et al., 2018; Park et al., 2019). The participants and personnel of three studies were not blinded to the combined intervention because of the intervention design's characteristics, which were considered to have a high risk of bias (Park, 2017; Donnezan et al., 2018; Park et al., 2019), while the outcome assessments of seven studies were blinded (Kounti et al., 2011; Hars et al., 2014; Lam et al., 2015; Rahe et al., 2015a; Delbroek et al., 2017; Park, 2017; Morita et al., 2018). A total of 13 studies showed a low risk of bias in attrition, reporting, and other biases (Fabre et al., 2002; Marmeleira et al., 2009; Shatil, 2013; Hars et al., 2014; Nishiguchi et al., 2015; Rahe et al., 2015a; Delbroek et al., 2017; Park, 2017; Morita et al., 2018; Mrakic-Sposta et al., 2018; Park et al., 2019, 2020; Rojasavastera et al., 2020).

For global cognitive function, the GRADE ratings from the included studies showed the effectiveness of “moderate” and “low” using the MMSE and MoCA to measure outcome (Table 1).

**Table 1:** Summary of the GRADEpro.

**Question:** Effects of combined intervention in the global cognition for older adults with MCI. **Setting:** Hospitals in mainland China **Intervention:** combined group **Comparison:** control group				
**Outcome measure**	**No of studies**	**No of the participants**	Anticipated absolute effects^*^ **(95% CI)**	**Certainty of the evidence (GRADE)**
MMSE	4	305	SMD 0.81 higher (0.51 higher to 1.11 higher)	⊕⊕⊕○ Moderate^a^
MoCA	4	95	SMD 0.93 higher (0.12 lower to 1.98 higher)	⊕⊕○○ Low^a^, ^b^
**Certainty of the evidence (GRADE)** High: We are very confident that the true effect lies close to that of the estimate of the effect. Moderate: We are moderately confident in the effect estimate; The true effect is likely to be close to the estimate of the effect, but there is a possibility that it is substantially different. Low: Our confidence in the effect estimate is limited: The true effect may be substantially different from the estimate of the effect. Very low: We have very little confidence in the effect estimate: The true effect is likely to be substantially different from the estimate of effect.				

* *The risk in the intervention group (and its 95% CI) is based on the assumed risk in the comparison group and the relative effect of the intervention (and its 95% CI).*

*CI, confidence interval; MMSE, the Mini-Mental State Examination; SMD, standardized mean difference; MoCA, the Montreal Cognitive Assessment.*

a *Most of the RCTs were low quality with an inadequate level of blinding and unclear risk of concealment of allocation.*

b *The statistical test for heterogeneity showed that large variation (I^2^ > 50%) existed in point estimates due to the among study differences*.

### Effects of the combined intervention

#### Effects of combined intervention in cognitively healthy older adults

##### Global cognition

**Figure 3:**
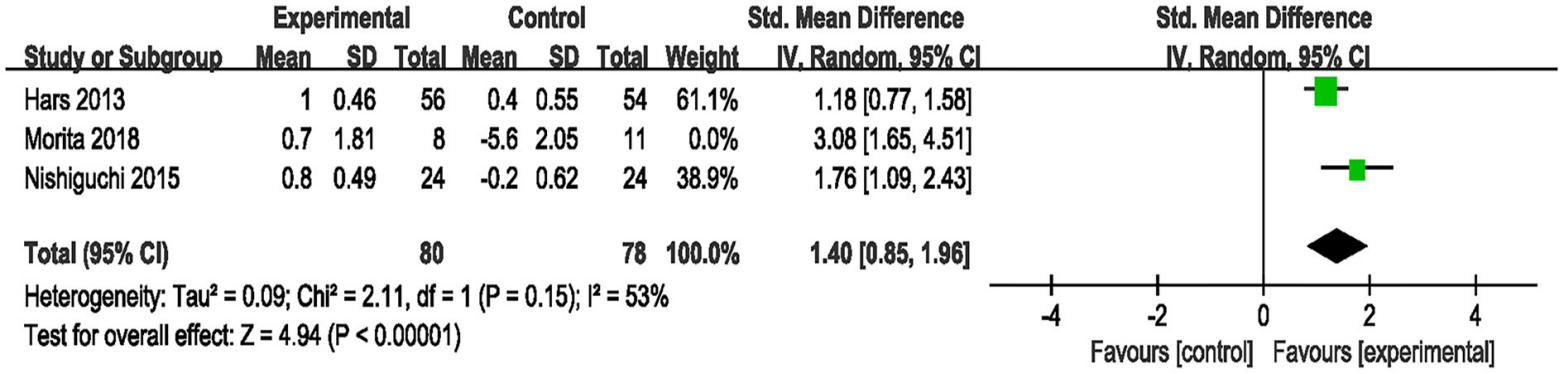
Forest plot of the efficacy of the combined intervention on global cognition in cognitively healthy older adults compared to the control group (sensitive analysis).

Three studies used MMSE to assess the efficacy of the combined intervention on global cognition in cognitively healthy older adults (Hars et al., 2014; Nishiguchi et al., 2015; Morita et al., 2018). Application of the RE model to the pooled SMD revealed that the global cognitive level was significantly higher in the combined group than in the control group (SMD = 1.77, 95% CI 0.94–2.59, *p* < 0.0001, Supplementary Figure S1). Next, due to the high heterogeneity (*I*^2^ = 73%, χ^2^ = 7.53, *p* = 0.02), we excluded one study at a time to perform sensitivity analysis. The result after excluding one study (Morita et al., 2018) showed the heterogeneity decreased (*I*^2^ = 53%, χ^2^ = 2.11, *p* = 0.15), as well as a change in the overall pooled effect (SMD = 1.40, 95% CI 0.85–1.96, *p* < 0.00001, Figure 3).

##### Cognition domains

**Figure 4:**
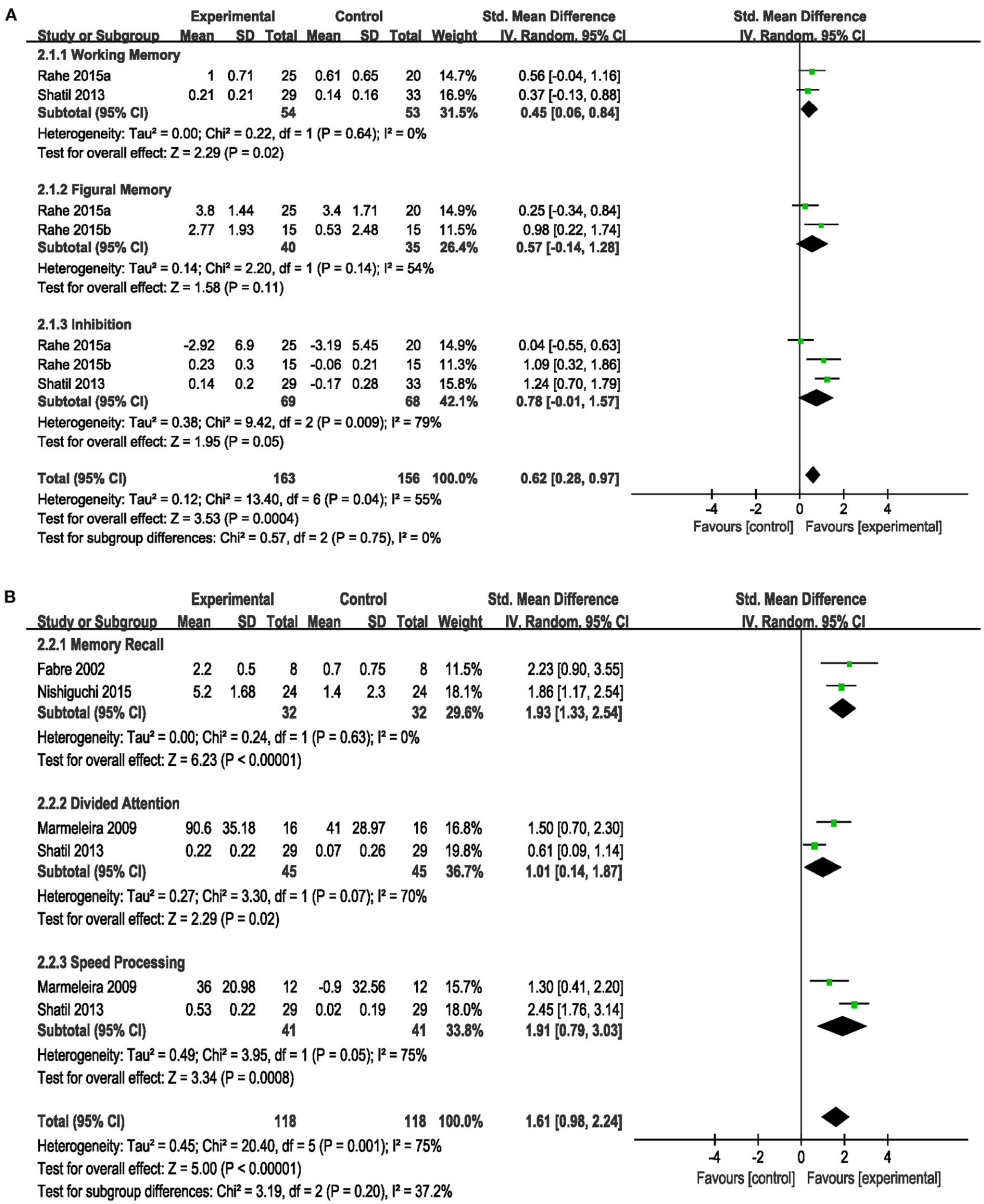
Forest plot of the efficacy of the combined intervention on cognition domains in cognitively healthy older adults. **(A)** Combined intervention vs. single cognitive intervention, **(B)** combined intervention vs. sham intervention.

Based on different cognition domains, we performed a subgroup analysis that compared the efficacy of the combined intervention with single cognitive, sham interventions to improve cognition in cognitively healthy older adults. Compared with single cognitive intervention (Figure 4A), the pooled SMD showed that combined intervention significantly improved working memory (SMD = 0.45, 95% CI 0.06–0.84, *p* = 0.02), but no significant improvement in figural memory (SMD = 0.57, 95% CI −0.14–1.28, *p* = 0.11) and inhibition (SMD = 0.78, 95% CI −0.01–1.57, *p* = 0.05). Compared with the sham intervention (Figure 4B), the combined intervention significantly improved memory recall (SMD = 1.93, 95% CI 1.33–2.54, *p* < 0.00001), divided attention (SMD = 1.01, 95% CI 0.14–1.87, *p* = 0.02) and speed processing (SMD = 1.91, 95% CI 0.79–3.03, *p* = 0.0008). However, this subgroup analysis showed a significant heterogeneity (*I*^2^ = 75%, χ^2^ = 20.40, *p* = 0.001), and we did not perform sensitivity analysis to identify the heterogeneity sources because of the limited number of studies in each subgroup. Different cognitive rating scales, intervention frequency, and duration may have contributed to the observed heterogeneity.

#### Effects of combined intervention in older adults with MCI

##### Global cognition

**Figure 5:**
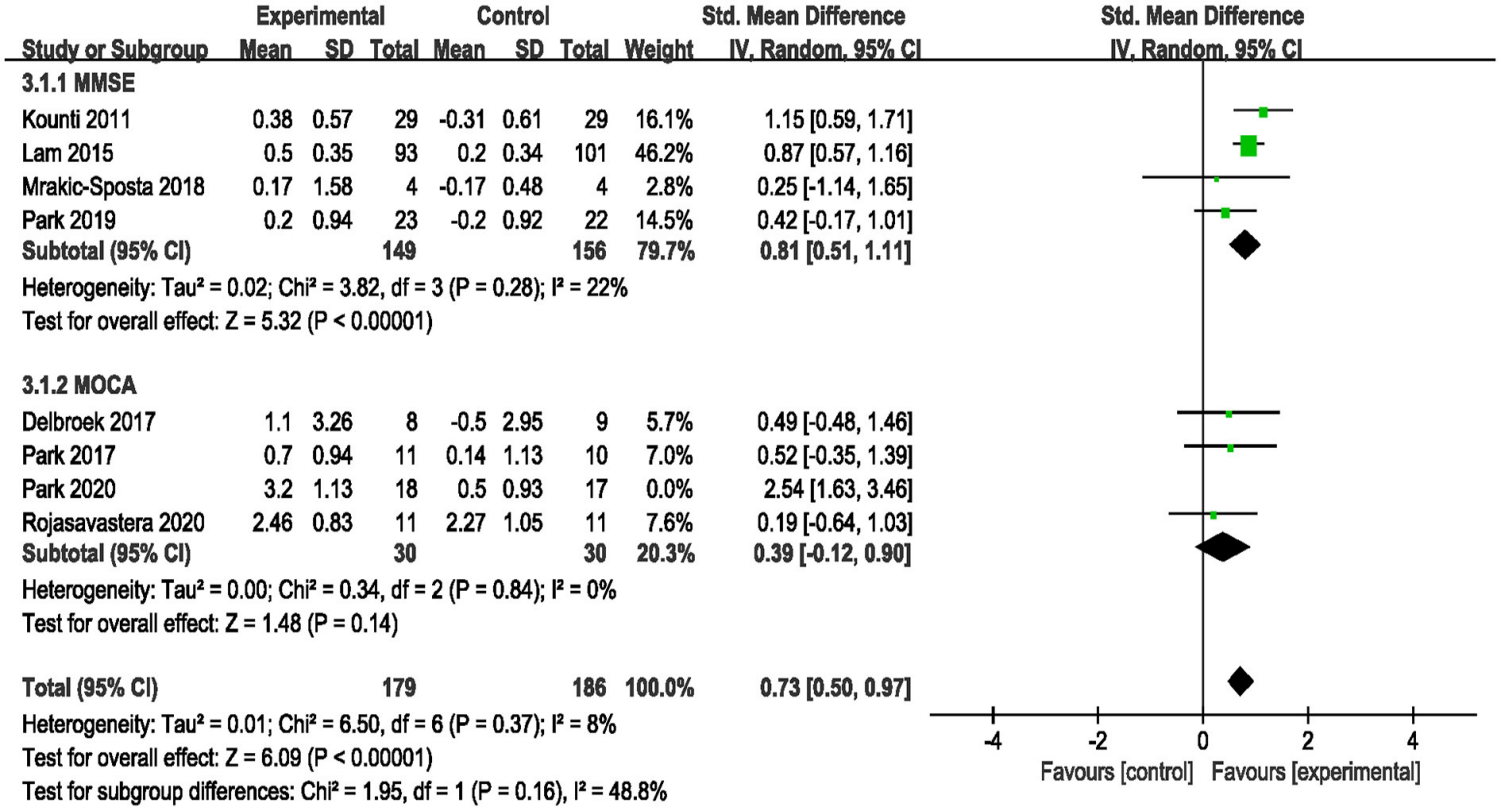
Forest plot of the efficacy of the combined intervention on global cognition in older adults with MCI compared to the control group (sensitive analysis).

Eight studies assessed the efficacy of the combined intervention on global cognition using the MMSE and MoCA (Kounti et al., 2011; Lam et al., 2015; Delbroek et al., 2017; Park, 2017; Mrakic-Sposta et al., 2018; Park et al., 2019, 2020; Rojasavastera et al., 2020). In a subgroup analysis based on different cognitive scales, the pooled SMD showed that combined intervention was more beneficial for improving global cognition (SMD = 0.83, 95% CI 0.41–1.25, *p* = 0.0001, Supplementary Figure S2). We performed a sensitivity analysis due to the high heterogeneity (*I*^2^ = 66%, χ^2^ = 20.39, *p* = 0.005). After excluding one study (Park et al., 2020), the heterogeneity decreased (*I*^2^ = 8%, χ^2^ = 6.50, *p* = 0.37), and the pooled result also changed (SMD = 0.73, 95% CI 0.50–0.97, *P* < 0.00001, Figure 5).

##### Cognition domains

**Figure 6:**
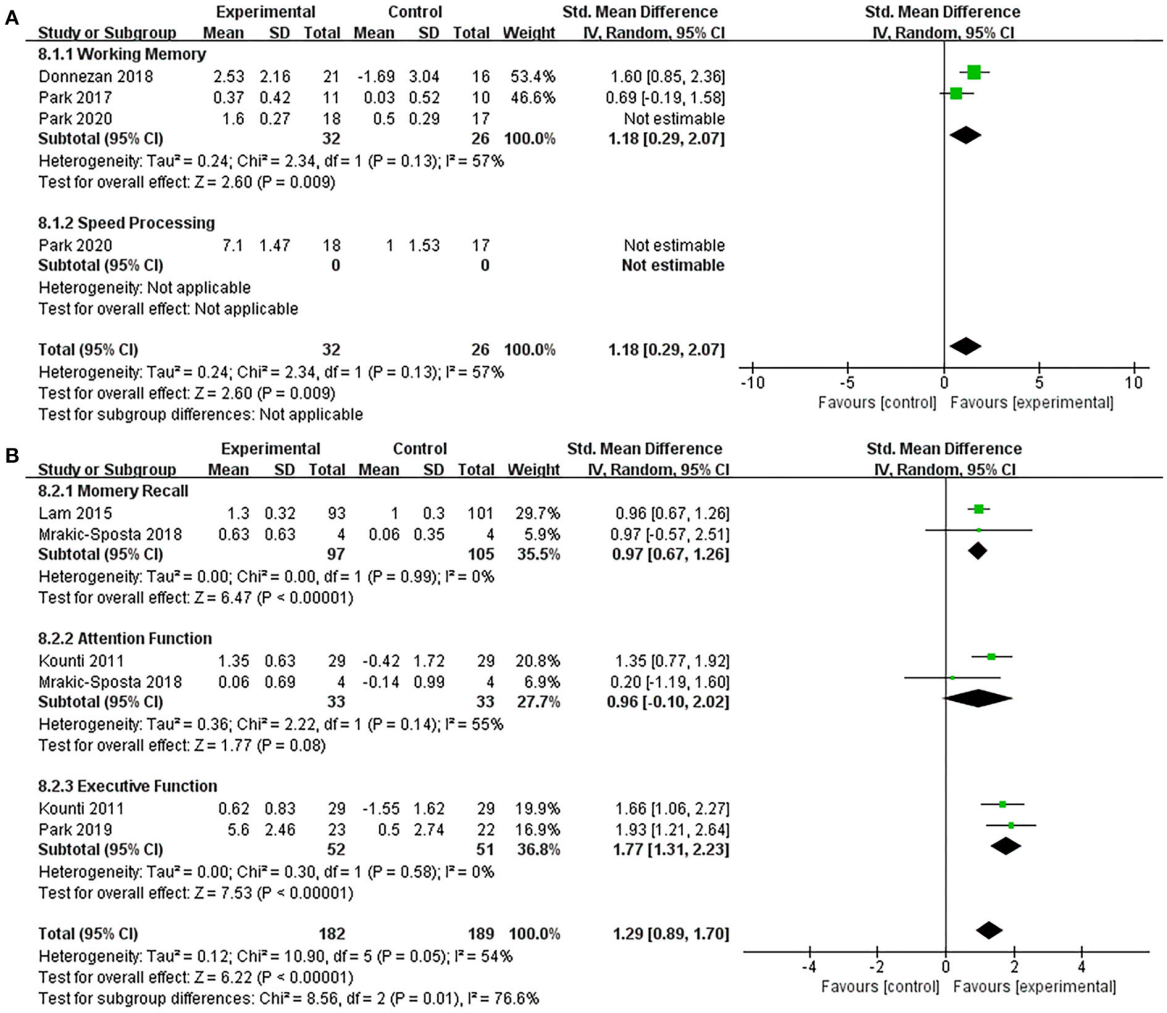
Forest plot of the efficacy of the combined intervention on cognition domains in older adults with MCI. **(A)** Combined intervention vs. single cognitive intervention (sensitive analysis), **(B)** combined intervention vs. sham intervention.

Subgroup analysis compared the efficacy of the combined intervention with single cognitive, sham intervention to improve cognition in older adults with MCI. Compared with the single cognitive intervention (Supplementary Figure S3), the results showed that combined intervention significantly improved working memory (SMD = 2.00, 95% CI 0.40–3.60, *p* = 0.01) and speed processing (SMD = 3.98, 95% CI 2.78–5.17, *p* < 0.00001). When we performed a sensitivity analysis due to the high heterogeneity (*I*^2^ = 90%, χ^2^= 29.43, *p* < 0.00001), the heterogeneity decreased (*I*^2^ = 57%, χ^2^ = 2.34, *p* = 0.13) after excluding one study (Park et al., 2020), and the overall pooled effect in working memory also changed (SMD = 1.18, 95% CI 0.29–2.07, *p* = 0.009, Figure 6A). Additionally, compared with the sham intervention (Figure 6B), under acceptable heterogeneity (*I*^2^ = 54%, χ^2^ = 10.90, *p* = 0.05), the subgroup analysis revealed that combined intervention significantly improved memory recall (SMD = 0.97, 95% CI 0.67–1.26, *p* < 0.00001) and executive function (SMD = 1.77, 95% CI 1.31–2.23, *p* < 0.00001), but no significant improvement in attention (SMD = 0.96, 95% CI −0.10–2.02, *p* = 0.08).

##### Depression

**Figure 7:**
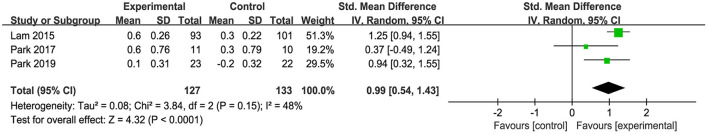
Forest plot of the efficacy of the combined intervention on depression in older adults with MCI compared with the control group.

Only three studies assessed the efficacy of the combined intervention to improve depression in older adults with MCI, with one study using CSDD (Lam et al., 2015) and two studies using GDS (Park, 2017; Park et al., 2019). Under acceptable heterogeneity (*I*^2^ = 48%, χ^2^ = 3.84, *p* = 0.15), the pooled results showed that combined intervention induced a significant improvement in depression (SMD = 0.99, 95% CI 0.54–1.43, *p* < 0.0001, Figure 7).

#### Efficacy differences of combined intervention between cognitively healthy older adults and older adults with MCI

As shown in Table 2, in order to reduce heterogeneity, we used the same comparison and outcome assessment scales to analyze the efficacy differences of the combined intervention in older adults with and without MCI. Therefore, the number of studies included was limited. After sensitivity analysis, the subgroup analysis showed that there were no statistical difference within the combined intervention to improve global cognition (SMD = 1.40, 95% CI 0.85–1.96, *p* < 0.00001; vs. SMD = 0.81, 95% CI 0.51–1.11, *p* < 0.00001), memory (SMD = 0.70, 95% CI 0.18–1.23, *p* = 0.009; vs. SMD = 1.18, 95% CI 0.29–2.07, *p* = 0.009), attention (SMD = −0.04, 95% CI −0.60–0.51, *p* = 0.88; vs. SMD = −0.08, 95% CI −0.94–0.78, *p* = 0.85), and executive function (SMD = 0.39, 95% CI −0.42–1.20, *p* = 0.35; vs. SMD = 0.62, 95% CI −0.07–1.30, *p* = 0.08) between cognitive healthy older adults and older adults with MCI.

**Table 2:** Efficacy differences of combined intervention on cognition between cognitively healthy older adults and older adults with MCI after sensitive analysis.

**Outcomes**	**Subgroup**	**No. of studies**		**SMD** **95% CI**	**Homogeneity**				**Test for overall** **effect**		**Test for** **subgroup^1, 2^** **differences**	
					***Q***	***df***	***p***	***I*** **^2^,%**	***Z***	***p***	***p***	***I*** **^2^,%**
Global cognition^a^	Subgroup^1^	3	1.40	0.85 to 1.96	2.11	1	0.15	53	4.94	<0.00001	0.07	70.2
	Subgroup^2^	4	0.81	0.51 to 1.11	3.82	3	0.28	22	5.32	<0.00001		
Memory^b^	Subgroup^1^	2	0.70	0.18 to 1.23	0.89	1	0.34	0	2.61	0.009	0.36	0
	Subgroup^2^	3	1.18	0.29 to 2.07	2.34	1	0.13	57	2.60	0.009		
Attention^c^	Subgroup^1^	1	−0.04	−0.60 to 0.51	NA	NA	NA	NA	0.15	0.88	0.94	0
	Subgroup^2^	2	−0.08	−0.94 to 0.78	NA	NA	NA	NA	0.19	0.85		
Executive function^d^	Subgroup^1^	2	0.39	−0.42 to 1.20	NA	NA	NA	NA	0.94	0.35	0.67	0
	Subgroup^2^	2	0.62	−0.07 to 1.30	1.20	1	0.27	16	1.77	0.08		

*SMD, standardized mean difference; CI, confidence interval; Subgroup^1^, the cognitively healthy older adults group; Subgroup^2^, the older adults with MCI group; NA, not applicable.*

a *Results of a study excluded after sensitivity analysis (Morita et al., 2018).*

b *Results of a study excluded after sensitivity analysis (Park et al., 2020).*

c *Results of a study excluded after sensitivity analysis (Donnezan et al., 2018).*

d *Results of a study excluded after sensitivity analysis (Nishiguchi et al., 2015)*.

#### The maintenance and safety of combined intervention

**Figure 8:**
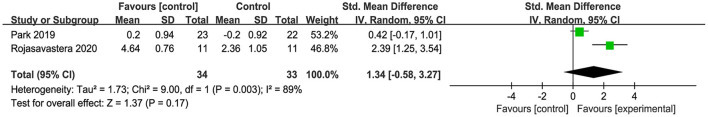
Forest plot of the maintenance on global cognition in older adults with MCI compared with sham intervention.

As shown in Figure 8, only two studies were included to assess the maintenance of the combined intervention on global cognition in older adults with MCI compared to the sham intervention (Park et al., 2019; Rojasavastera et al., 2020), and the results showed no statistical difference (SMD = 1.34, 95% CI −0.58–3.27, *p* = 0.17). Similarly, due to limited follow-up data, we did not perform a subgroup analysis based on the different cognitive scales, which may have been a source of the observed high heterogeneity.

The minor adverse event was the risk of falls in older adults while performing physical training. The researchers increased safety protection and education for older adults to minimize this risk.

#### Moderator analysis for combined intervention

As shown in Table 3, because the outcome assessment scales and comparisons were not fully the same among studies, we only assessed the effect of the moderator variables on the efficacy of the combined intervention in order to improve global cognition in older adults with MCI. The results of the subgroup analyses showed that age (SMD = 0.73, 95% CI −0.21–1.66, *p* = 0.13; vs. SMD = 0.74, 95% CI 0.49–0.99, *p* < 0.00001), education (SMD = 0.75, 95% CI 0.49–1.01, *p* < 0.00001; vs. SMD = 0.73, 95% CI −0.21–1.66, *p* = 0.13), intervention duration (SMD = 0.37, 95% CI −0.1–0.85, *p* = 0.13; vs. SMD = 0.79, 95% CI 0.08–1.511, *p* = 0.03) and the mode of combined intervention (SMD = 0.69, 95% CI 0.35–1.03, *p* < 0.0001; vs. SMD = 0.65, 95% CI 0.03–1.27, *p* = 0.04) had an effect on the efficacy of the combined intervention in improving cognition. However, we were unable to draw a precise conclusion about whether intervention frequency affected the efficacy of the combined intervention because there was only one study with an intervention frequency more than 3 days per week.

**Table 3:** Effects of moderators on the efficacy of combined intervention to improve cognition in older adults with MCI after sensitive analysis.

**Moderator variable**	**Level (subgroup)**	**No. of studies**	**SMD**	**95% CI**	**Homogeneity**				**Test for overall** **effect**		**Test for subgroup** **differences**	
					***Q***	***df***	***p***	***I** ^**2**^* **, %**	***Z***	***p***	***p***	***I** ^**2**^* **, %**
Age^a^^,^^c^	≤ 70 years	2	0.73	−0.21 to 1.66	3.49	1	0.06	71	1.52	0.13	0.97	0
	>70years	5	0.74	0.49 to 0.99	2.54	3	0.47	0	5.81	<0.00001		
Education^b^^,^^c^	Elementary school	4	0.75	0.49 to 1.01	2.04	2	0.36	2	5.67	<0.00001	0.96	0
	Middle to high school	2	0.73	−0.21 to 1.66	3.49	1	0.06	71	1.52	0.13		
Intervention duration^c^	≤ 3 months	5	0.37	−0.11 to 0.85	0.37	3	0.95	0	1.52	0.13	0.23	32.5
	3–6 months	2	0.79	0.08 to 1.51	3.10	1	0.08	68	2.18	0.03		
	>6 months	1	0.87	0.57 to 1.16	NA	NA	NA	NA	5.76	<0.00001		
Mode of combined intervention^c^	Simultaneous	6	0.69	0.35 to 1.03	4.11	4	0.39	3	3.99	<0.0001	0.90	0
	Sequential	2	0.65	0.03 to 1.27	2.21	1	0.14	55	2.06	0.04		

*SMD, standardized mean difference; CI, confidence interval; NA, not applicable.*

a *One study was excluded because the mean age of participants was not reported (Park, 2017).*

b *Two studies was excluded because education level was not reported (Delbroek et al., 2017; Mrakic-Sposta et al., 2018).*

c *Results of a study excluded after sensitivity analysis (Park et al., 2020)*.

## Discussion

### Summary of findings

#### Global cognition

The results of our analysis showed that the combined intervention group was superior to the control group in improving global cognition in older adults with and without MCI, which is consistent with the results of other studies (Karssemeijer et al., 2017; Gavelin et al., 2021). Dual or multi-tasking training of combined cognitive and physical intervention is the basis to improve global cognition and ADL, which can reduce neurophysiological changes in cognition by reducing bilateral prefrontal cortical oxygenation, increasing hippocampal volume, and increasing white matter integrity (Tait et al., 2017). However, due to the limited number of studies, we did not perform subgroup analyzes according to different comparison conditions in global cognition. Additionally, seven studies assessed global cognition by MMSE (Kounti et al., 2011; Hars et al., 2014; Lam et al., 2015; Nishiguchi et al., 2015; Delbroek et al., 2017; Park, 2017; Morita et al., 2018), but two of them (Lam et al., 2015; Morita et al., 2018) using modified MMSE, which may limit the credibility of the results, so the results should be interpreted carefully. This also emphasizes the necessity on further evaluate the specific cognition domains to draw accurate conclusions.

#### Cognition domains

There is growing evidence that even the aging brain displays cognitive plasticity (Park and Bischof, 2013; Pauwels et al., 2018). Yang et al. (2020) reported that combined intervention improved most cognitive function in older adults with and without MCI, but had no effect on attention, and it was uncertain whether these positive effects would persist (Yang et al., 2020), which is consistent with our findings. Based on the theory of dual-task interference, the superior effect of the combined intervention may not be observed in the short term because of the cognitive and physical interaction. Therefore, the follow-up assessments are critical when studying the efficacy of the combined intervention to improve cognition in older adults in the future.

#### Depression

Based on the pathophysiological mechanisms of cognitive deficits and depression, we found an apparent correlation between them (Geda et al., 2006; Pellegrino et al., 2013), In older adults with MCI, patients with depression ranged from 20.1 to 44.3% (Panza et al., 2010). The statistical results of a study showed a positive correlation between the severity of depression and MCI, with depression significantly affecting delayed recall, verbal fluency, attention, and executive function in older adults (Dillon et al., 2009). Furthermore, depression as a risk factor for MCI has significant public health implications. Our results revealed that combined intervention had a small to moderate positive effect on depression, and other studies have reported that improvements in depression reduce the severity of MCI (Kessing et al., 2011; Pellegrino et al., 2013). A study by Barnes and Yaffe (2011) reported that a 10% reduction in depression prevalence could lead to 326,000 fewer AD cases worldwide.

#### Efficacy differences of combined intervention between cognitively healthy older adults and older adults with MCI

Our review reported that there was no statistical difference in the efficacy of the combined intervention for improving cognition in older adults with and without MCI, which is inconsistent with the findings of Wu et al. (2019), who suggested that the combined intervention was more effective in improving global cognition in older adults with MCI compared to cognitively healthy older adults (Wu et al., 2019). We used the same comparison and outcome assessment scales to assess efficacy differences, resulting in a limited number of studies included for this outcome; therefore, the results should be interpreted cautiously.

#### The maintenance and safety of combined intervention

Due to limited follow-up data, this meta-analysis only reported that the efficacy of the combined intervention in improving global cognition in older adults with MCI was not maintained (Park et al., 2019; Rojasavastera et al., 2020); however, another three studies found positive maintenance of the combined intervention (Barnes et al., 2013; Lee et al., 2016; Norouzi et al., 2019). In summary, we found heterogeneity primarily in two areas: the types of physical tasks within the combined intervention and the modes of the combined intervention. Regarding the types of physical task, resistance training (Norouzi et al., 2019), combined aerobic and resistance training (Barnes et al., 2013; Lee et al., 2016) improved the long-term working memory and global cognition within older adults with MCI; however, aerobic training alone was not found to have positive efficacy maintenance (Park et al., 2019; Rojasavastera et al., 2020). Thus far, combined aerobic and resistance training is the most commonly used and effective type of exercise (Kelly et al., 2014). Furthermore, the modes of combined intervention are divided into sequential (Park et al., 2019; Rojasavastera et al., 2020) and simultaneous interventions (Barnes et al., 2013; Norouzi et al., 2019). It was found that simultaneous intervention is superior to sequential intervention during efficacy maintenance, which may be based upon the mechanisms of physical-cognitive interaction. This result validates the intervention mode derived in our review as an influential factor in the efficacy of the combined intervention and is also consistent with the results of other meta-analyses (Zhu et al., 2016). However, it remains controversial whether the time of each sequential intervention is the same as that of simultaneous intervention (Joubert and Chainay, 2018).

Except for a slight risk of falls, none of the included studies reported significant adverse events during the combined intervention. Furthermore, due to the limited sample size, the safety and maintenance of the combined intervention will need to be validated *via* multicenter studies with larger sample sizes, and more follow-ups.

#### Moderators analysis for combined intervention

In terms of demographic characteristics, this review found that age and education level were influential factors in the efficacy of the combined intervention. Moreover, the combined intervention was more effective during advanced age as well as less educated older adults, which may be related to this population's lower baseline cognitive performance. Previous studies found a positive association between age and the efficacy of the combined intervention, while no correlation was reported in education (Powers et al., 2013; Toril et al., 2014; Qarni and Salardini, 2019).

Different intervention durations also affected the efficacy of the combined intervention. Law et al. (2014) found that an intervention duration of 3–6 months was more beneficial for improving cognition in older adults with MCI (Law et al., 2014), and is consistent with the results of our study. Suzuki et al. (2012) also reported that a 6-month combined intervention effectively improved cognition in older adults; however, the efficacy did not last until the end of the 12 month treatment regimen. Due to the limited number of included studies, we were unable to draw a precise conclusion about whether intervention frequency affected the efficacy of the combined intervention. However, a previous meta-analysis found that high-frequency combined intervention might be ineffective (Zhu et al., 2016). Two studies on working memory also reported that high-frequency intervention might lead to cognitive fatigue causing participants to drop out of the study (Penner et al., 2012; Wang et al., 2014). In conclusion, selecting the appropriate intervention frequency and duration is likely to be an essential factor in improving the efficacy of a combined intervention.

### Limitations

This meta-analysis also has some limitations. First, the number of included studies was limited. Second, the outcome measurements did not use imaging, electroencephalogram (EEG), or other objective evaluation methods. The evidence suggests structural and functional magnetic resonance imaging or electrophysiological measurements of brain activity can more accurately evaluate the changes of specific areas in the brain (Bherer et al., 2013). Third, only English articles were included.

### Implications for future studies

Two points need to be improved in the future. First, to maximize the effect of intervention, future studies need to stringently design the mode, frequency, and duration of the combined intervention, and a long-term follow-up. Second, we need to select more appropriate outcome measurement indexes, comprehensive neuropsychological assessments, and objective evaluation tools (e.g., imaging and EEG) to accurately assess the efficacy of the combined intervention.

## Conclusion

In summary, this meta-analysis showed that combined cognitive and physical intervention effectively improves cognition in older adults with and without MCI compared with single cognitive or sham intervention, although the intervention effects vary by cognition domains. However, it is challenging to draw an obvious conclusion in the combined intervention maintenance because of the limitations. Additionally, there was no statistical difference in the efficacy of the combined intervention to improve cognition between cognitive healthy older adults and older adults with MCI. The results should be interpreted carefully due to the different intervention designs and the diversity of evaluation methods. In the future, more stringent study designs with more follow-ups are needed to clarify the effects of the combined intervention and provide guidance on the optimum intervention regime for improving cognitive function in older adults.

## Data availability statement

The original contributions presented in the study are included in the article/Supplementary Material, further inquiries can be directed to the corresponding author/s.

## Author contributions

KH contributed to study design, literature search, figures, data extraction, data analysis, and writing. ZT contributed to literature search, data extraction, and data analysis. ZB and WS contributed to figures, data extraction, data interpretation, and writing. HZ contributed to study design and data interpretation. All authors contributed to the article and approved the submitted version.

## Funding

This work was supported by the National Key Research and Development Program of China (Grant No. 2018YFC2001703), Capital Health Research and Development of Special Fund (Grant No. 2020-1-6011), and China Rehabilitation Research Center Key Project (Grant No. 2021ZX-02).

## Conflict of interest

The authors declare that the research was conducted in the absence of any commercial or financial relationships that could be construed as a potential conflict of interest.

## Publisher's note

All claims expressed in this article are solely those of the authors and do not necessarily represent those of their affiliated organizations, or those of the publisher, the editors and the reviewers. Any product that may be evaluated in this article, or claim that may be made by its manufacturer, is not guaranteed or endorsed by the publisher.
