# Supplementary material for: Effects of combined cognitive and physical intervention on enhancing cognition in older adults with and without mild cognitive impairment: A systematic review and meta-analysis
Source: Front Aging Neurosci. 2022 Jul 19;14:878025. doi: 10.3389/fnagi.2022.878025 (PMC9343961; doi:10.3389/fnagi.2022.878025)
Supplement: Supplementary file 1 [file Data_Sheet_1.doc]

Supplementary Material

**1 Supplementary Tables**

Table S1 | Supplementary search strategy

| Number | Search strategy |
| --- | --- |
| #1 | “cognitive training” OR “cognitive intervention” OR “memory training” OR “attention training” OR “execution training” |
| #2 | “physical training” OR “aerobic training” OR “resistance training” OR “exercise*” OR “balance*” OR “walk*” OR “gait” |
| #3 | #1 and #2 |
| #4 | elderly OR aging OR aged OR old* OR "age groups" OR seni* OR retire* OR geriatric |
| #5 | “combined” OR “combination” OR “dual task*” OR “dual-task*” OR “double task*” OR “two task*” OR “secondary task*” OR “second task*” OR “additional task*” OR “multiple task*” OR “multi-modal” |
| #6 | #3 and #4 and #5 |

Table S2 | Characteristics of included studies.

| **Study** | **Country** | **Population**  **diagnosis** | **Number** | **Men**  **(%)** | **age**  **(y)** | **Education**  **(y or %)** | **Intervention design** | | | | **Treatment condition** | | **Outcome measure** | **Follow-up** | **Drop out** | **PEDro**  **score** |
| --- | --- | --- | --- | --- | --- | --- | --- | --- | --- | --- | --- | --- | --- | --- | --- | --- |
|  |  |  |  |  |  |  | **Experimental** | | **Control** | | **Frequency** | **Duration** |  |  |  |  |
| Fabre  et al. (2002) | France | Cognitively healthy | CCPI:8  SI:8 | 12.5 | CCPI: 64.9±1.4  SI: 65.7±1.5 | CCPI: 12.10±1.20  SI: 12.10±1.40 | Dual-tasks of combined aerobic and memory training | sequential | | sham intervention  (no-therapy) | 60-90min/d，3d/week | 8 weeks | ①Memory Recall: WMS | NA | CCPI: 0  SI: 0 | 7 |
| Hars  et al.  (2014) | Switzerland | Cognitively healthy | CCPI:56  SI:54 | 3.73 | CCPI:  75.00±8.00  SI:  76.00±6.00 | Primary school：14.93%  Middle school:  67.16%  High school: 17.91% | Multi-tasks of physical training(walking) and cognitive training (handling objects,quick reactions) following music | simultaneous | | sham intervention  (no-therapy) | 60min/d,  1d/week | 25weeks | ①Global Cognition: MMSE | NA | CCPI: 10  SI: 14 | 8 |
| Marmeleira et al.  (2009) | Portugal | Cognitively healthy | CCPI:16  SI:16 | 78.13 | CCPI:  68.2±6.5  SI: 68.4±6.7 | CCPI: 4.8±3.1  SI: 5.1±2.2 | Multi-tasks of physical abilities and cognitive, perceptive training | simultaneous | | sham intervention  (no-therapy) | 60min/d, 3d/week | 12weeks | ①Divided attention: SCWT  ②Speed processing: TMT | NA | CCPI: 0  SI: 0 | 8 |
| Morita et al. (2018) | Japan | Cognitively healthy | CCPI:8  SI:11 | 10.5 | CCPI:  75.0±1.5  SI:  71.9±4.0 | CCPI:  11.6±1.0  SI: 12.7±1.9 | Dual tasks of cognitive-motor training | simultaneous | | sham intervention  (non-  exercise) | 60min/d, 1d/week | 2years | ①Global Cognition: MMSE | NA | CCPI: 0  SI: 0 | 7 |
| Nishigu  chi et al.  (2015) | Japan | Cognitively healthy | CCPI:24  SI:24 | 54.17 | CCPI:  73.0±4.8  SI:  73.5±5.6 | CCPI:  12.2±2.2  SI: 13.0±2.5 | Dual tasks–based multimodal training | simultaneous | | sham intervention  (no-therapy) | 90min/d, 1d/week | 12weeks | ①Global Cognition: MMSE  ②Memory Recall: WMS | NA | CCPI: 0  SI: 0 | 8 |
| Rahe  et al.  (2015a) | Germany | Cognitively healthy | CCPI:25  SCI:20 | 37.78 | CCPI:  68.44±7.36  SCI:  67.65±6.86 | CCPI:  14.44±3.34  SCI:  14.80±2.82 | Dual tasks of cognitive training with additional physical activity | sequential | | Single cognitive training | 90min/d, 2d/week | 7weeks | ①Working memory:  WAIS-DST  ②Attention function:  BTA  ③Inhibition: SCWT  ④Verbal fluency: RWT | NA | CCPI: 3  SCI: 3 | 7 |
| Rahe  et al.  (2015b) | Germany | Cognitively healthy | CCPI:15  SCI:15 | 30 | CCPI:  67.13±4.09  SCI:  66.33±5.33 | CCPI:  16.87±4.22  SCI:  14.27±4.08 | Dual tasks of cognitive training with additional physical activity | sequential | | Single cognitive training | 110min/d, 2d/week | CCPI:  6.5weeks  SCI:6  weeks | ①Figural memory:  CFT  ②Attention function: BTA  ③Executive control/ Inhibition: TMT  ④Verbal fluency: COWA | 1year | CCPI: 9  SCI: 0 | 7 |
| Shatil  et al.  (2013) | USA | Cognitively healthy | CCPI:29  SCI:33  SI:29 | 31.87% | CCPI:  79±5.49  SCI:  80±5.43  SI:81±5.25 | college and above:72.53% | Dual tasks of cognitive training and physical activity | sequential | | SCI:Single cognitive training  SI:  sham intervention  (reading) | 40-45min/d,  3d/week | 16weeks | ①Working memory: CogniFit  ②Divided attention:  CogniFit  ③Inhibition: CogniFit  ④Speed processing: CogniFit | NA | CCPI: 19  SCI: 12  SI: 13 | 7 |
| Delbroek et al. (2017) | Belgium | MCI | CCPI:8  SI:9 | 35 | CCPI:  86.9 ± 5.6  SI:  87.5 ± 6.6 | NA | Dual tasks of VR-based cognitive-motor training | simultaneous | | sham intervention (no additional training) | 18min/d,2d/week,1week;30min/d ,2d/week,5week | 6weeks | ①Global Cognition: MoCA | NA | CCPI: 2  SI: 1 | 9 |
| Donnezan et al.  (2018) | France | MCI | CCPI:20  SCI:14 | NA | CCPI: 75.2±1.3  SCI：76.3±1.5 | CCPI：5.9±0.31  SCI：5.5±0.36 | Dual tasks of aerobic training on bikes and cognitive training (commercialized gaming software “HAPPYneuron” and Presco) | simultaneous | | Single cognitive training | 60min/d, 2d/week | 12weeks | ①Working memory: DST | 6months | CCPI: 1  SCI: 5 | 7 |
| Kounti  et al.  (2011) | Greek | MCI | CCPI:29  SI:29 | 20.69 | CCPI: 70.48±7.52  SI: 67.83±7.29 | CCPI: 9.59±4.77  SI: 7.79±3.79 | RHEA intervention | simultaneous | | sham intervention  (no-therapy) | 90min/d, 1d/week | 20weeks | ①Global Cognition: MMSE  ②Executive function: FUCAS  ③Attention function: TEA | NA | CCPI: 20  SI: 10 | 7 |
| Lam  et al.  (2015) | China | MCI | CCPI:93  SI:101 | 21.67 | CCPI: 76.3±6.6  SI: 75.4±6.1 | CCPI: 3.4±3.3  SI: 4.0±3.9 | Dual tasks of one cognitive and two types of mind body exercises | sequential | | sham intervention  (social activity) | CCPI:1h/d, 3d/week  SI: ≥3 sessions/  week | 1year | ①Global Cognition: MMSE  ②Momery recall: WAIS  ③Depression: CSDD | NA | CCPI: 39  SI: 30 | 8 |
| Mrakic-  Sposta  et al.  (2018) | Italy | MCI | CCPI:4  SI:4 | 40 | CCPI: 72.00±5.15  SI: 74.60±6.43 | NA | Dual tasks of VR-based program combining aerobic exercise and cognitive training | simultaneous | | sham intervention  (no-therapy) | 40-45min/  session, 3sessions/  week | 6weeks | ①Global Cognition: MMSE  ②Momery recall: RAVLT  ③Attention function: AM | NA | CCPI: 1  SI: 1 | 7 |
|  |  |  |  |  |  |  |  |  | |  |  |  |  |  |  |  |
| Park  et al.  (2017) | Korea | MCI | CCPI:11  SCI:10 | 28.57 | ≥ 60 | CCPI: 7.00±3.28  SCI: 6.33±3.81 | Dual tasks of cognitive training with exercise program | simultaneous | | Single cognitive or exercise training | 60min/d, 2d/week | 8weeks | ①Global Cognition: MoCA  ②Working memory: WAIS-DST  ③Depression: GDS | NA | CCPI: 0  SCI: 0 | 9 |
| Park  et al.  (2019) | Korea | MCI | CCPI:23  SI:22 | 30.61 | CCPI: 70.55±6.46 SI: 72.76±5.37 | CCPI: 7.15±2.94  SI: 7.05±3.28 | Dual tasks of cognitive training with physical activity,aerobic exercise | simultaneous | | sham intervention  (no-therapy) | 110min/  session,  1session/  week | 24weeks | ①Global Cognition: MMSE  ②Exucutive function: SDST  ③Depression: GDS | 3months | CCPI: 2  SI: 2 | 8 |
| Park  et al.  (2020) | Korea | MCI | CCPI:18  SCI:17 | 48.57 | CCPI: 75.8±8.5  SCI: 77.2±7.2 | Uneducated:  2/1  Eelementary School: 13/13  Middle School: 2/2  High school: 1/1  University: 0/0 | Dual tasks of VR-based cognitive-motor rehabilitation usinng MOTOcog | simultaneous | | conventional  cognitive rehabilitation | 30min/d, 5d/week | 6weeks | ①Global Cognition: MoCA  ②Speed processing: TMT  ③Working memory: DST | NA | CCPI: 2  SCI: 3 | 8 |
| Rojasavastera  et al.  (2020) | Thailand | MCI | CCPI:11  SI:11 | 22.73 | CCPI: 67.64±4.64  SI: 65.71±2.45 | CCPI: 13.50±3.25  SI: 12.73±4.43 | Dual tasks of action observation abilities with gait training | sequential | | sham intervention  (no-therapy) | 65min/  session, 2-3sessions/  week, | 4-6  weeks | ①Global Cognition: MoCA | 1month | CCPI: 2  SI: 2 | 7 |

CCPI, combined cognitive and physical intervention; SCI, single cognitive intervention; SI, sham intervention; MMSE, Mini-Mental State examination; MoCA, Montreal Cognitive Assesstment; TMT, Trail Making Test; WMS, Wechsler Memory Scale; WAIS-DST, Wechsler Adult Intelligence Scale Digit Span Test; SDST, Symbol-Digit Substitution Test; COWA, Controlled Oral Word Association Test; SCWT, Stroop color word test; RAVLT, Recall of Rey Auditory Verbal Learning Test; BTA,Brief Test of Attention; RWT, Regensburger Wort Flüssigkeits-Test; CFT, Complex Figure Test; AM, Attentional Matrices; TEA, Test of Everyday Attention; CogniFit, the CogniFit neuropsychological evaluation; GDS, Geriatric Depression Scale; CSDD, Cornell Scale for Depression in Dementia; FUCAS, Executive Function Cognitive Assessment Scale;

**2 Supplementary Figures**

FIGURE S1 | Forest plot of the efficacy of the combined intervention on global cognition in cognitively healthy older adults compared to the control group.

FIGURE S2 | Forest plot of the efficacy of the combined intervention on global cognition in older adults with MCI compared to the control group.

FIGURE S3 | Forest plot of the efficacy of the combined intervention on cognition domains in older adults with MCI. (A) combined intervention versus single cognitive intervention, (B) combined intervention versus sham intervention.
