# Supplementary figures and images for: Effects of combined cognitive and physical intervention on enhancing cognition in older adults with and without mild cognitive impairment: A systematic review and meta-analysis
Source: Front Aging Neurosci. 2022 Jul 19;14:878025. doi: 10.3389/fnagi.2022.878025 (PMC9343961; doi:10.3389/fnagi.2022.878025)

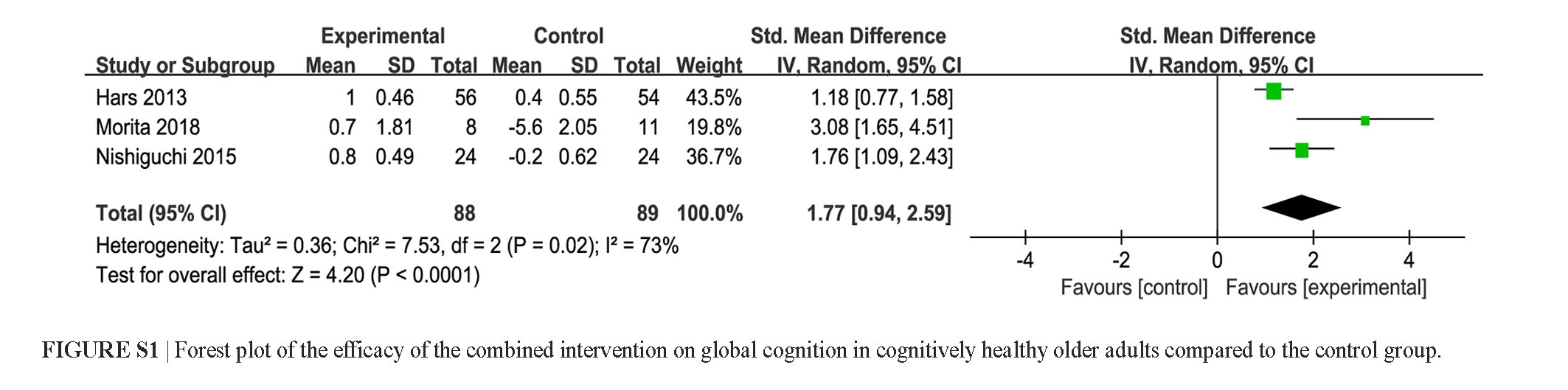

Supplement: Supplementary file 2 [file Image_1.TIF]

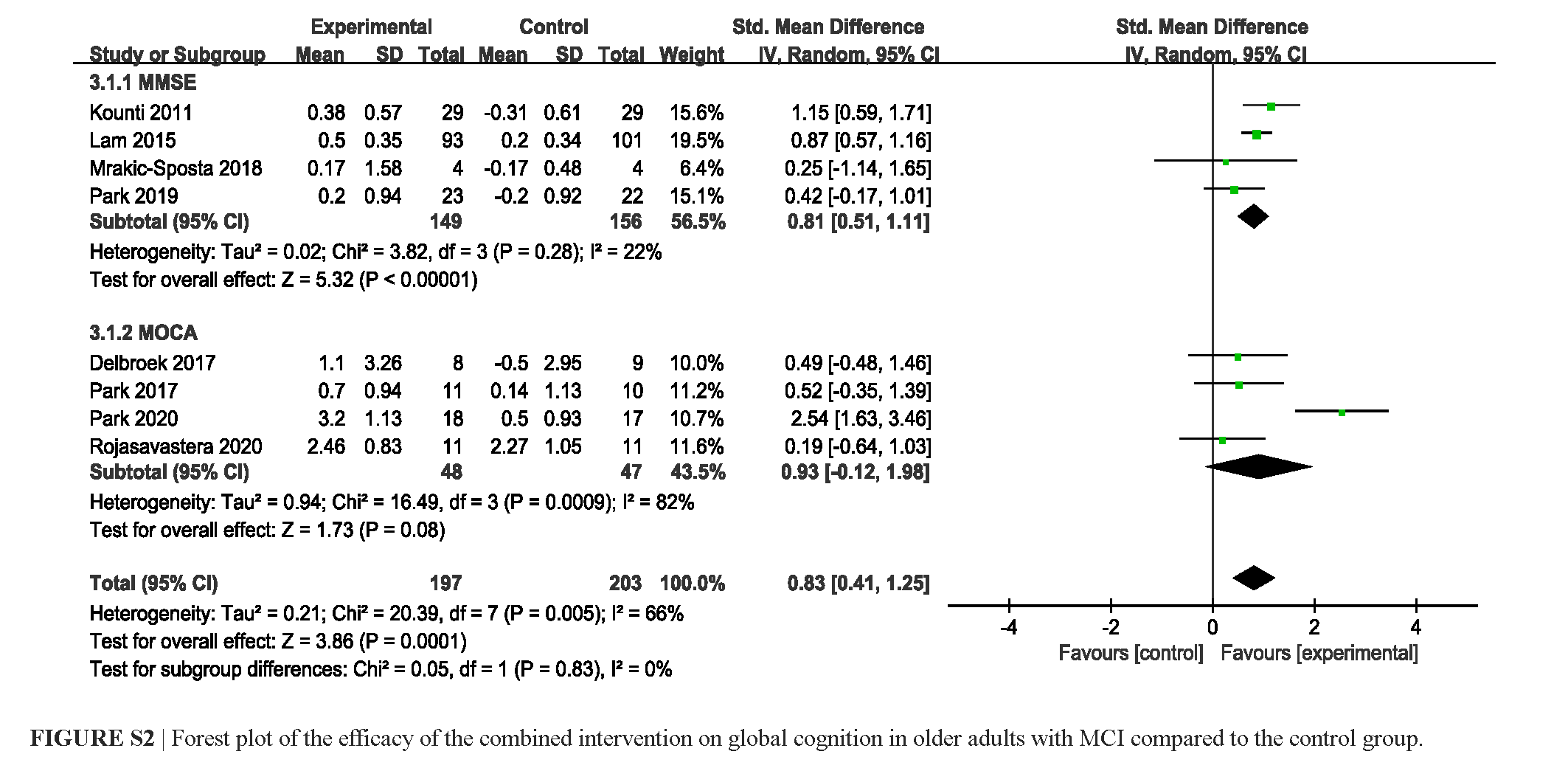

Supplement: Supplementary file 3 [file Image_2.TIF]

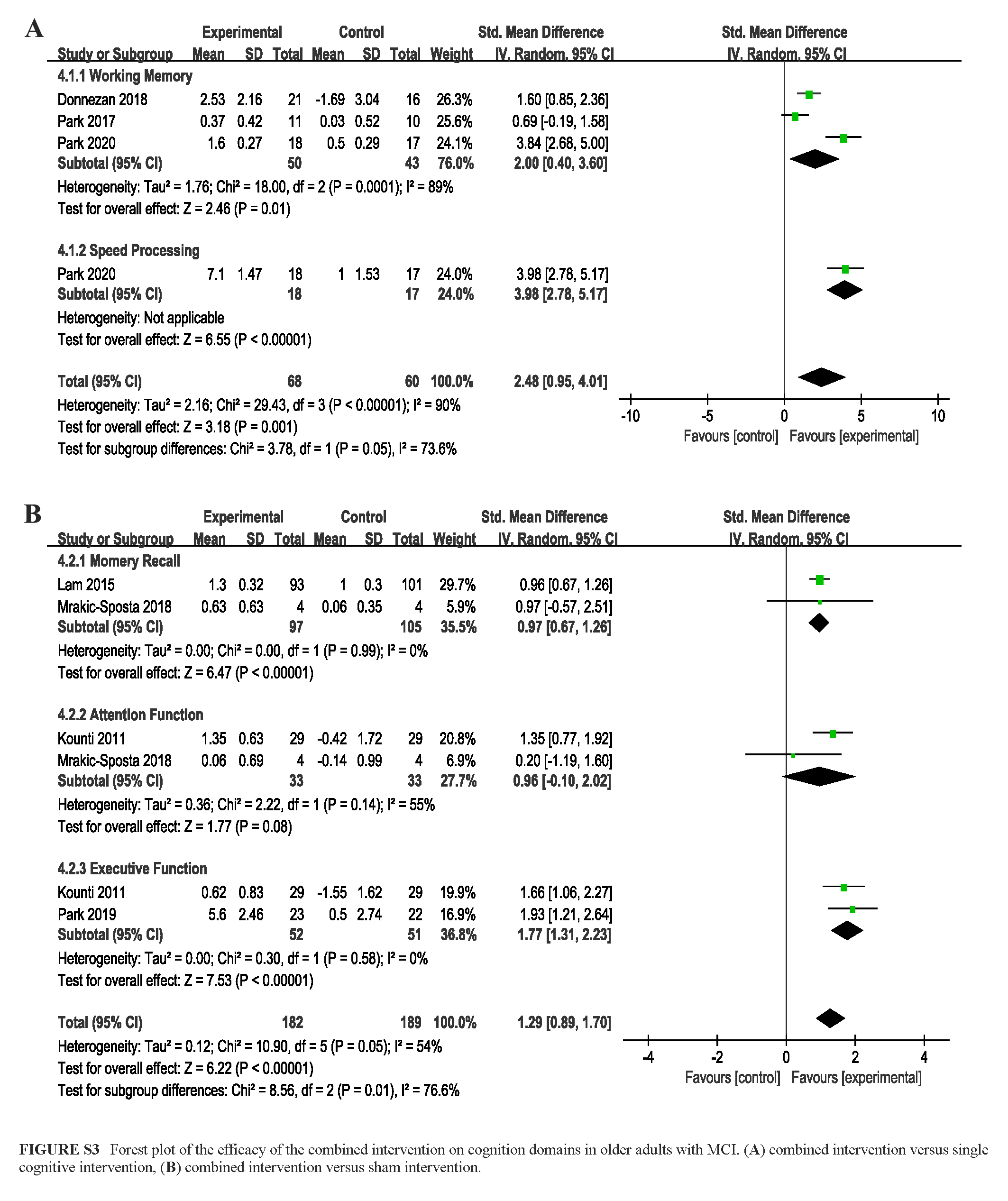

Supplement: Supplementary file 4 [file Image_3.TIF]
